# Neglected Anterior Shoulder Dislocation With Greater Tuberosity Fracture Managed With Open Reduction and Latarjet Procedure: A Case Report

**DOI:** 10.7759/cureus.60333

**Published:** 2024-05-15

**Authors:** Mainak Roy, Suhas Aradhya Bhikshavarthi Math, Vivek Tiwari, Samir Dwidmuthe

**Affiliations:** 1 Orthopaedics, All India Institute of Medical Sciences, Nagpur, Nagpur, IND; 2 Orthopaedic Surgery, All India Institute of Medical Sciences, Nagpur, Nagpur, IND; 3 Orthopaedics, Apollo Sage Hospital, Bhopal, IND

**Keywords:** surgical management, latarjet procedure, open reduction, greater tuberosity fracture, neglected shoulder dislocation

## Abstract

Neglected shoulder dislocation is a relatively rare occurrence characterized by structural changes in bone and soft tissue. Surgical intervention is often necessary, yet no universally accepted treatment approach exists, presenting a challenging clinical scenario.

A 45-year-old female presented with an eight-month-old neglected anterior shoulder dislocation, compounded by a Hill-Sachs lesion from a previous fall. Treatment comprised open reduction in conjunction with the Latarjet procedure. Regular follow-up evaluations were conducted over three years post-surgery, revealing satisfactory outcomes including good range of motion, bony union, and absence of dislocation episodes.

Managing neglected shoulder dislocations, particularly those with significant bone defects, poses unique challenges involving soft tissue contracture, bone loss, and associated fractures. Despite these complexities, open reduction combined with the Latarjet procedure demonstrated a high success rate in preventing further shoulder dislocation, albeit with a persistent risk of shoulder joint osteoarthritis.

## Introduction

Glenohumeral joint dislocation stands as the most common type of joint dislocation, with anterior dislocations comprising approximately 95% of cases, making them notably more frequent than posterior dislocations [1]. Neglected shoulder dislocations, though rare, often involve structural changes in bones and soft tissues, necessitating extensive surgical intervention [2]. Isolated displaced greater tuberosity fractures, occurring in less than 2% of proximal humeral fractures, are typically associated with anterior shoulder dislocations. The greater tuberosity fragment is pulled superiorly by the supraspinatus and posteriorly by the infraspinatus and teres minor [3]. There is no established standard treatment for chronic neglected anterior shoulder dislocation, posing challenges for patients and clinicians alike. Treatment decisions predominantly rely on lower-tier studies and literature focusing on recurrent dislocations, given the scarcity of high-tier studies directly comparing various treatment modalities. Here, we present the case of an eight-month-old neglected anterior shoulder dislocation with fractured greater tuberosity managed with open reduction and Latarjet procedure.

## Case presentation

A 45-year-old woman sought medical attention at our hospital due to a left shoulder deformity that had persisted for the past eight months. Her injury occurred after she slipped in the bathroom and fell on her shoulder. After the incident, she experienced intense pain and a noticeable deformity in her left shoulder. Initially, she pursued treatment from a bone setter in an attempt to alleviate her discomfort and restore normal function to her shoulder. However, the pain and deformity persisted. In an attempt to immobilize the injured shoulder and reduce pain, she wore an arm sling for approximately three months. Over time, she gradually regained some limited movement in her left shoulder, though it was not adequate to carry out her daily activities.

The examination findings of the patient's left shoulder indicated significant abnormalities. Notably, there was a loss of normal shoulder contour, along with an empty glenoid fossa suggestive of dislocation. A globular mass was observed inferior to the coracoid process, moving with the humerus shaft. Deltoid atrophy was evident, and Duga's test yielded a positive result. Range of motion (ROM) assessment revealed severe limitations: flexion was restricted to 0-30 degrees, abduction to 0-20 degrees, and internal rotation to 0-10 degrees, with no external rotation observed. Interestingly, the "regimental badge" sign was negative.

Diagnostic imaging

**Figure 1:**
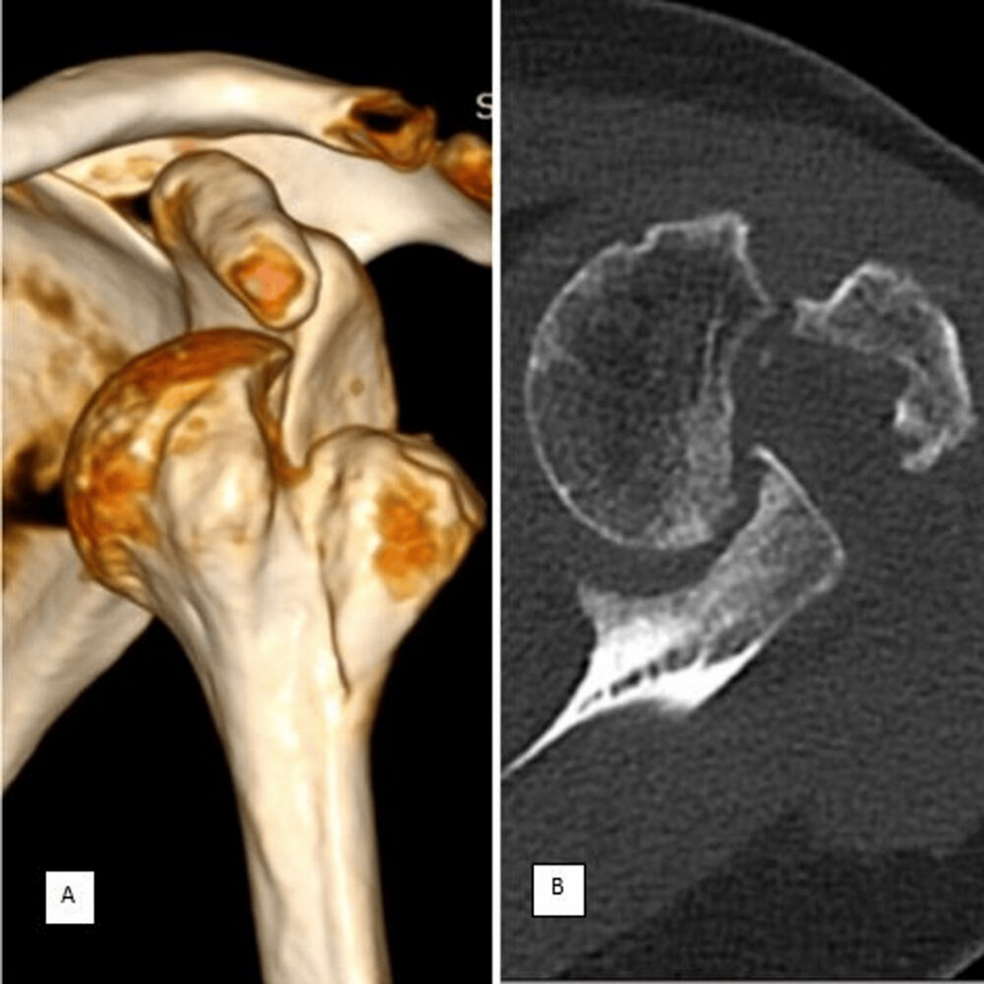
(A) 3D CT image showing locked anterior shoulder dislocation. (B) CT image showing >40% Hill-Sachs lesion on the posterior aspect of the humerus head

The X-ray of the patient's left shoulder showed a fracture of the greater tuberosity along with anterior dislocation, classified as sub-coracoid type. The CT scan (Figure 1) confirmed the presence of a large Hill-Sachs lesion (>40%) and locked anterior shoulder dislocation with a glenoid bone loss of >25%, indicating significant joint instability and malpositioning.

**Figure 2:**
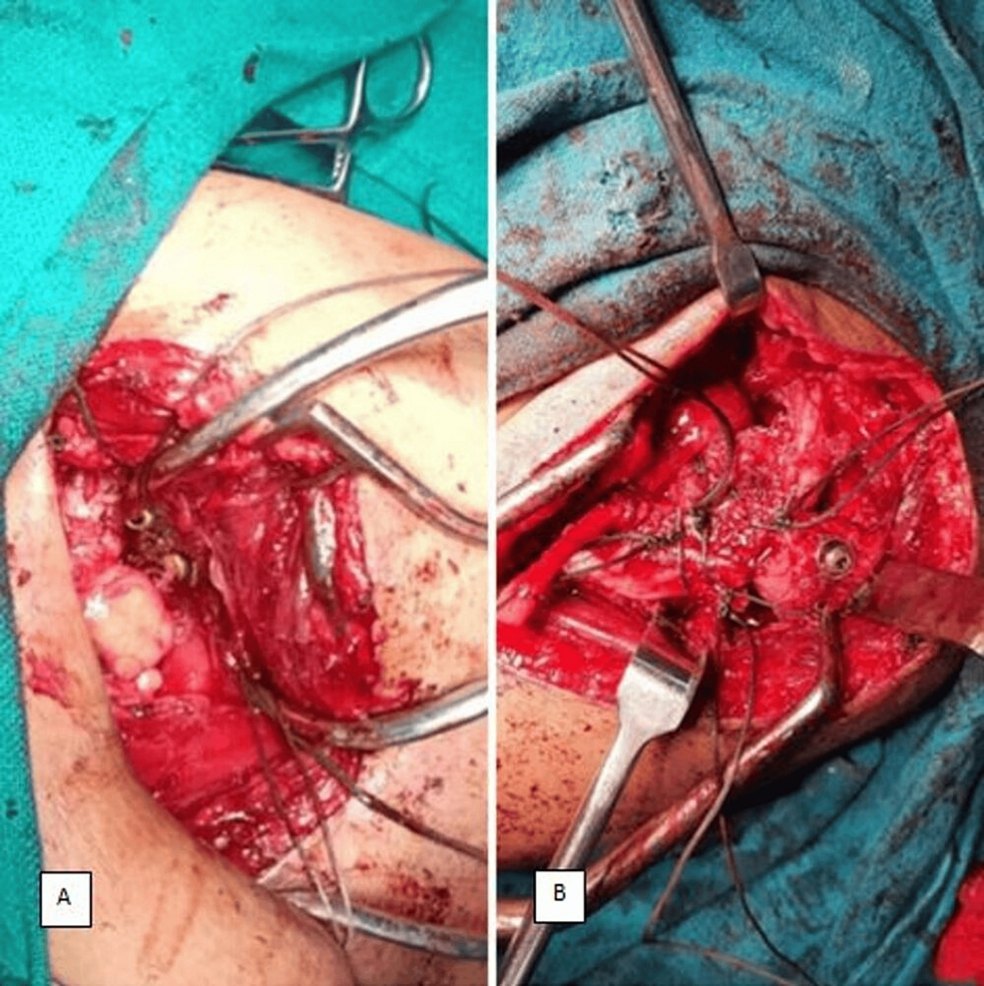
Intraoperative image showing (A) the Latarjet procedure (transfer of the coracoid process to the anteroinferior aspect of the glenoid) and (B) fixation of the greater tuberosity fragment

We opted for an open reduction and anterior shoulder stabilization with Latarjet procedure and greater tuberosity fixation with cannulated cancellous (CC) screws. Extensive fibrotic tissue was encountered intraoperatively around the shoulder joint, necessitating meticulous removal to facilitate the reduction process. Subsequently, the Latarjet procedure was performed, involving the transfer of the coracoid process along with the conjoint tendon to the anteroinferior aspect of the glenoid, where it was securely fixed in place with CC screws. The greater tuberosity was fixed in its original place with CC screws (Figure 2).

**Figure 3:**
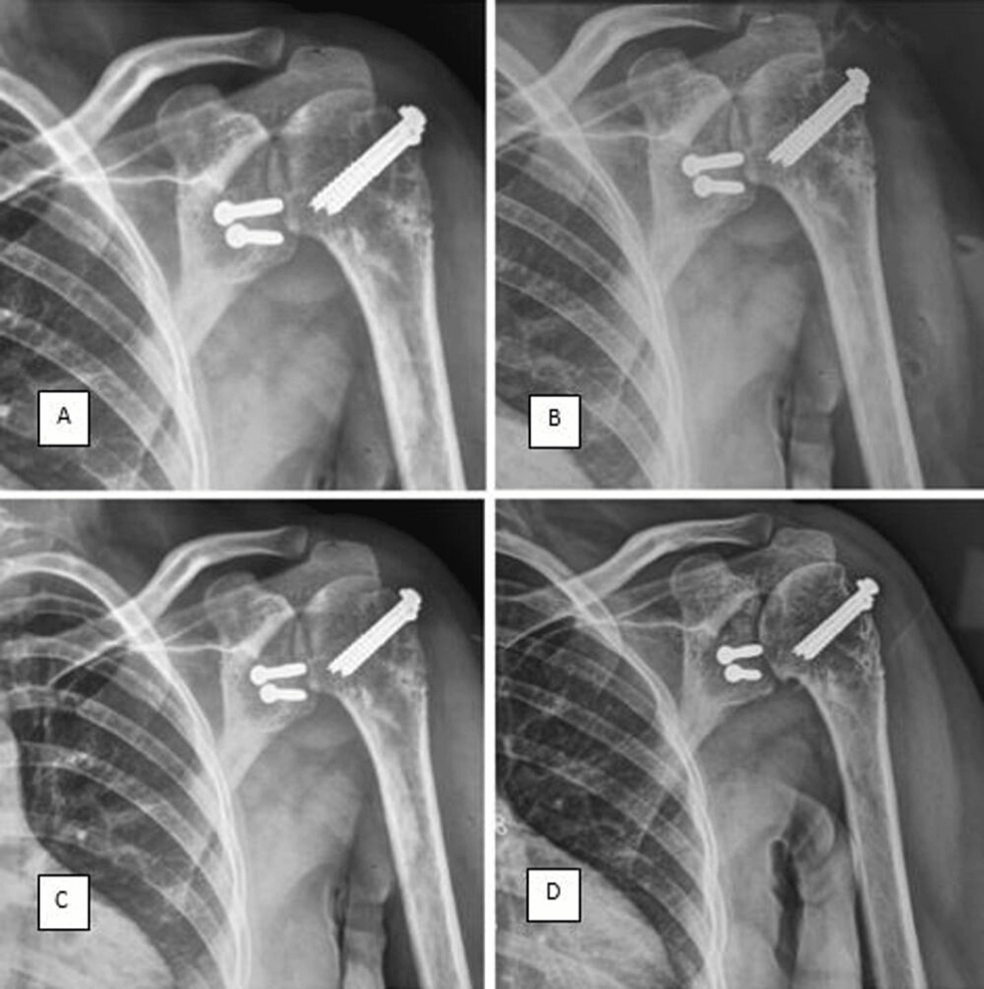
Anteroposterior views of sequential postoperative X-rays taken at (A) four months, (B) nine months, (C) two years, and (D) three years, showing no signs of glenohumeral subluxation and well-positioned implants

Postoperative X-rays confirmed the successful reduction of the shoulder joint (Figure 3).

The patient was advised to wear a shoulder immobilizer and a chest binder to maintain the arm in the adducted position, ensuring stability and support during the initial recovery period. After suture removal, Codman pendular exercises were initiated to facilitate gentle movement and prevent stiffness in the shoulder joint. As the patient progressed in her recovery, passive rotation exercises and shoulder range of motion exercises were introduced three weeks post-surgery, with the intensity adjusted according to pain tolerance. After six weeks, active range of motion exercises were started to further improve shoulder mobility and strength. These postoperative measures were carefully tailored to promote a smooth and successful recovery while minimizing the risk of complications.

**Figure 4:**
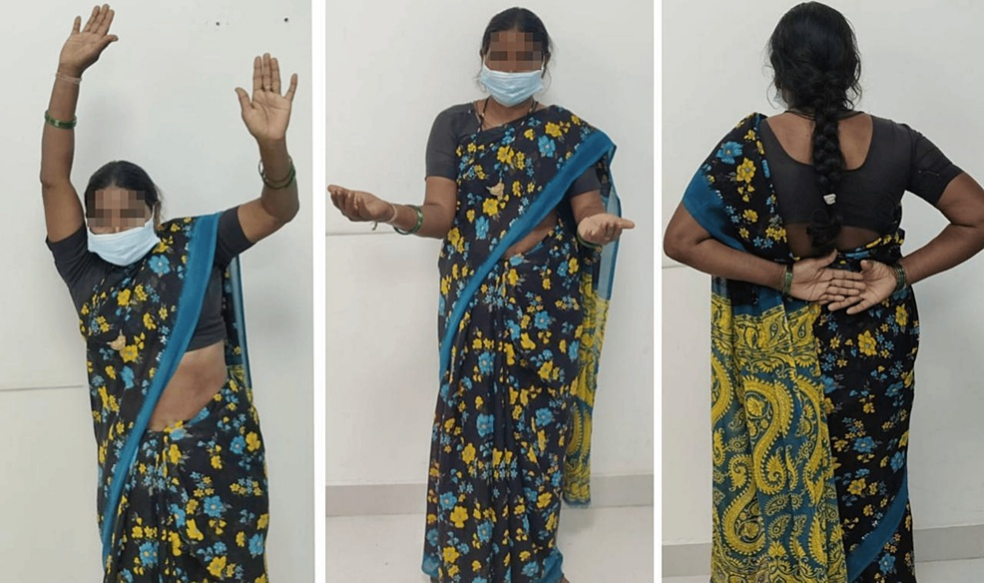
Three-year postoperative follow-up clinical image showing satisfactory range of motion in her left shoulder

The patient was closely followed up at regular intervals for three years postoperatively (Figure 4). She had a satisfactory recovery with a good range of motion; only her shoulder abduction was limited (0-100°). She was able to perform most of her daily activities efficiently. After her surgery, she had no history of any repeat dislocation.

## Discussion

The glenohumeral joint stands out as the most commonly dislocated joint in the body, with anterior dislocations comprising 95% of all shoulder dislocations. Trauma, particularly when the arm is abducted and extended, is the primary mechanism for unilateral injuries, leading to anterior shoulder dislocation as the greater tuberosity abuts against the acromion, causing leverage forces that dislodge the humeral head from the glenoid cavity [1]. Chronic shoulder dislocation refers to cases where injury recognition is delayed for at least three or four weeks, often accompanied by pathological changes in bone and soft tissue structures, necessitating extensive surgical intervention [4]. Neglected shoulder dislocation presents a challenging dilemma for both patients and clinicians, often resulting in severe complications such as Hill-Sachs and Bankart lesions, glenoid bone loss, rotator cuff tears, and eventual glenohumeral osteoarthritis [5]. Surgical management options include observation, closed reduction under anesthesia, open reduction, Bankart repair, capsulolabral repair, and arthroplasty [4]. Open reduction surgery is typically recommended for neglected dislocations for more than four weeks to mitigate the risk of further complications [2]. Bony defects result due to continuous friction between the dislocated humeral head and the anterior edge of the glenoid [4]. This friction can lead to recurrent instability, with the severity depending on the size and depth of the bony defect. When the defect falls between 25% and 40% of the glenoid surface, various surgical options are considered. Anatomic procedures, such as allograft reconstruction of the head or humeral head dis-impaction/humeroplasty, aim to restore the natural anatomy of the joint. Non-anatomic procedures are also prevalent, including osseous or soft tissue transfer (such as remplissage) and the Latarjet procedure. The Latarjet procedure, which involves the transfer of the coracoid process to the glenoid rim, is particularly favored for its "triple effect," which provides stability to the joint by addressing both bone and soft tissue deficiencies [6]. For defects exceeding 40-50% of the head, treatment options typically involve more extensive interventions. In younger patients, rotational proximal humeral osteotomy may be considered to address such substantial defects. Alternatively, partial or total humeral head arthroplasty may be recommended as viable options [4]. Studies have suggested that compared to soft tissue reconstructions like Bankart repair, an open Latarjet procedure is more effective in managing recurrent anterior shoulder dislocations associated with significant glenoid bone loss [7]. However, it's important to note that despite its efficacy, the Latarjet procedure has been associated with a notable incidence of complications. These include a high rate of redislocation or subluxation, as well as limitations in both external and internal rotation. Additionally, there have been reports of the deterioration or early onset of glenohumeral osteoarthritis following this procedure [8]. Both Haroun et al. and Cho et al. found that patients treated with Latarjet surgery had higher postoperative complications like coracoid nonunion, screw backout, and osteoarthritis [9,10]. Itoi had advised that patients involved in contact sports and those who require a full range of motion should be operated on by a Latarjet procedure [11]. The Latarjet procedure performed in this patient has effectively stabilized the shoulder joint following neglected dislocation, and not much complication was noted over the period of three years after the surgery.

## Conclusions

Managing neglected shoulder dislocations presents intricate challenges often necessitating surgical intervention. Our case study of a 45-year-old woman with an eight-month-old neglected anterior shoulder dislocation illustrates successful treatment with open reduction and the Latarjet procedure. At the end of the three-year post-surgical follow-up, she demonstrated satisfactory range of motion and bony union without recurrence of dislocation. While effective, the Latarjet procedure entails risks such as redislocation and osteoarthritis. Nevertheless, it conferred stability and functional enhancement in our case, underscoring its efficacy in addressing neglected shoulder dislocations. Further prospective, long-term studies are warranted to comprehensively evaluate outcomes and potential complications.
